# Metagenome-Assembled Genome Sequence of Stenotrophomonas maltophilia Strain UJ_SKK_5.5, Obtained from the Gut Microbiome of a Macrotermes bellicosus Termite Collected from Hot, Arid Nigeria

**DOI:** 10.1128/mra.01060-22

**Published:** 2023-01-04

**Authors:** Enoch B. Joel, Jessica L. Lenka, Bitrus Yakubu, Richard J. Kutshik, Aminu Tukur, Ishaya Y. Longdet

**Affiliations:** a Department of Biochemistry, University of Jos, Jos, Nigeria; b Department of Biochemistry and Drug Development, NVRI, Vom, Nigeria; c Quality Control Dept, Nigeria National Petroleum Corporation, Port Harcourt Refinery, Port Harcourt, Nigeria; University of Southern California

## Abstract

The metagenome-assembled genome (MAG) sequence of Stenotrophomonas maltophilia strain UJ_SKK_5.5 was obtained from the gut microbiome of Macrotermes bellicosus (termite) from hot, arid Nigeria. The assembled genome (4,313,335 bp) contains 157 contigs, the *N*_50_ is 41,072 bp, the GC content is 66.57%, and there are 3,925 protein coding sequences, 3,886 proteins with functional assignments, 39 pseudogenes, and 67 RNA genes.

## ANNOUNCEMENT

*Stenotrophomonas* species occur ubiquitously (1). They have biotechnological potentials including producing antimicrobial compounds, generating plant growth factors, bioremediation and phytoremediation (2–4), lignocellulose degradation (5), high resistance to heavy metals and antibiotics (6), and roles in nitrogen and sulfur cycles (1). Stenotrophomonas maltophilia is an emerging human pathogen (6). Here, we report the genome sequence of Stenotrophomonas maltophilia strain UJ_SKK_5.5 derived from the gut of a Macrotermes bellicosus termite.

The *Macrotermes bellicosus* termites were collected from Illela, Sokoto State, Nigeria (13.7273°N, 5.2972°E) in February 2021 and cleaned 3 times (dipped in 70% ethanol for 3 min and rinsed with sterile water). A termite’s gut was extracted and crushed in phosphate-buffered saline (PBS). Since our interest is in organisms with lignocellulose-degrading capacity, the crushed guts were cultured on plated medium prepared from kraft lignin, M9 salts, and agar in a ratio of 2:1:2 at 37°C for 72 h and subcultured in four successions on the same medium. We expected pure isolates, but Gram staining revealed a mixture of organisms with varied morphological features (cocci, rods, pairs, chains, or singles). To obtain cell pellets, the organisms (from one colony) were grown overnight in nutrient broth at 37°C. Two sets of 2-mL portions (from the overnight broth) were centrifuged at 14,000 × *g* for 3 min. The combined cell pellets were washed (in 500 μl of phosphate-buffered saline and centrifuged at 14,000 × *g* for 3 min) twice. DNA was extracted using the ZymoBiomics DNA Miniprep kit according to the manufacturer’s instructions.

DNA libraries were prepared using the Nextera XT DNA library preparation kit (Illumina) and the Nextera index kit (Illumina), and genomic DNA was fragmented using Illumina Nextera XT fragmentation enzyme. Twelve cycles of PCR were performed to construct libraries, which were purified using AMPure magnetic beads and eluted in Qiagen EB buffer. The libraries were quantified using a Qubit 4 fluorometer and a Qubit double-stranded DNA (dsDNA) high-sensitivity (HS) assay kit and sequenced on an Illumina HiSeq X platform with 2- by 150-bp read lengths, producing 11.321 million raw reads. The raw reads were trimmed and processed using Fastp v0.20.1 (7) with a cut mean quality of 15, assembled into contigs using MEGAHIT v1.0 (8), and then binned using MetaBAT2 v2.15 (9). These produced 1 metagenome-assembled genome (MAG) (ABHC2_5.5) with completeness of ≥50% and contamination of ≤10% (10). Here, default parameters were used for all software. Taxonomic classification, completeness, and contamination were assessed using QUAST v4.4 (11) and BUSCO v5 (12).

The ABHC2_5.5 genome has 157 contigs and the following characteristics: assembled size, 4,313,335 bp; completeness, 99.2%; fragmentation, 0.8%; missing portion, 0.00%; *N*_50_, 41,072 bp; GC content, 66.57%; BUSCO percent score, C:123 (S:123, D:0), F:1, M:0, n:124. The genome annotation using PGAP v6.1 (13) identified 3,925 protein coding sequences, 3,886 proteins with functional assignments, 39 pseudogenes, and 67 RNA genes.

**FIG 1:**
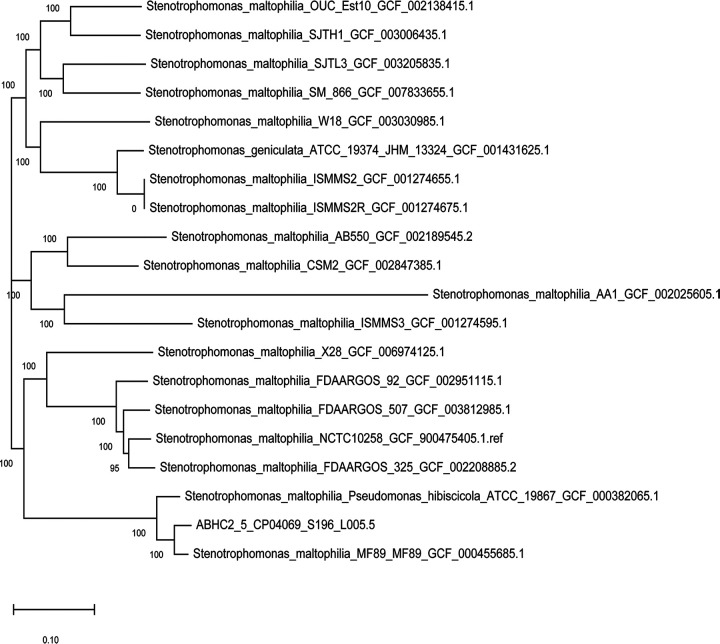
SNP tree based on ABHC2_5.5 core genome phylogeny.

To generate a single nucleotide polymorphism (SNP) tree (Fig. 1), assembled contigs were processed through the CosmosID core genome SNP typing pipeline to evaluate phylogenetic placement and SNP differences using Parsnp (14) as the core genome aligner, which reconstructed the phylogenomic relationship using FastTree2 (15). Closely related strains of Stenotrophomonas maltophilia were presented. Accordingly, ABHC2_5.5 was named Stenotrophomonas maltophilia strain UJ_SKK_5.5.

### Data availability.

The genome for Stenotrophomonas maltophilia strain UJ_SKK_5.5 has been deposited at DDBJ/EMBL/GenBank under accession number JANIOO000000000. The version described in this paper is version JANIOO010000000. The raw reads were deposited in the Sequence Read Archive (SRA) under accession number SRR21003156 and in BioSample under number SAMN28179101.
